# Linkage mapping of quantitative trait loci for fiber yield and its related traits in the population derived from cultivated ramie and wild *B. nivea* var. *tenacissima*

**DOI:** 10.1038/s41598-019-53399-5

**Published:** 2019-11-14

**Authors:** Zheng Zeng, Yanzhou Wang, Chan Liu, Xiufeng Yang, Hengyun Wang, Fu Li, Touming Liu

**Affiliations:** 1 Institute of Bast Fiber Crops and Center of Southern Economic Crops, Chinese Academy of Agricultural Sciences, Changsha, China; 2 Shanghai OE Biotech. Co., Ltd, Shanghai, China

**Keywords:** Genetic linkage study, Quantitative trait

## Abstract

Ramie is an important natural fiber crop, and the fiber yield and its related traits are the most valuable traits in ramie production. However, the genetic basis for these traits is still poorly understood, which has dramatically hindered the breeding of high yield in this fiber crop. Herein, a high-density genetic map with 6,433 markers spanning 2476.5 cM was constructed using a population derived from two parents, cultivated ramie Zhongsizhu 1 (ZSZ1) and its wild progenitor *B. nivea* var. *tenacissima* (BNT). The fiber yield (FY) and its four related traits—stem diameter (SD) and length (SL), stem bark weight (BW) and thickness (BT)—were performed for quantitative trait locus (QTL) analysis, resulting in a total of 47 QTLs identified. Forty QTLs were mapped into 12 genomic regions, thus forming 12 QTL clusters. Among 47 QTLs, there were 14 QTLs whose wild allele from BNT was beneficial. Interestingly, all QTLs in Cluster 10 displayed overdominance, indicating that the region of this cluster was likely heterotic loci. In addition, four fiber yield-related genes underwent positive selection were found either to fall into the FY-related QTL regions or to be near to the identified QTLs. The dissection of FY and FY-related traits not only improved our understanding to the genetic basis of these traits, but also provided new insights into the domestication of FY in ramie. The identification of many QTLs and the discovery of beneficial alleles from wild species provided a basis for the improvement of yield traits in ramie breeding.

## Introduction

Fibers are widespread among vascular plant species and are present in many different organs including roots, stems, and leaves. It plays important roles in the growth and development of plant, including establishing plant architecture, defending from herbivory, storing ergastic carbon resources and water^1^. In addition, plant fibers are one of the most important renewable resources, and are used as raw material in the paper industry, and for various textiles and for composites. Because the remarkable valuable of fiber for human and plant itself, the fiber traits have been paid wide attention in research, especially in *Populus* and cotton.

Ramie (*B. nivea* var. *nivea*), a diploid (2n = 28) perennial herbaceous plant, is one of the most important natural fiber crops in China, and has been cultivated with more than 4,700 years^2^. Ramie fibers are one of the longest fibers in plant kingdoms, and its length can reach to 55 cm^3^. Moreover, unlike *Populus* and cotton fibers that are xylem and seed epidermal fibers, respectively, ramie fiber are extracted from stem barks and are bast fibers. Although these interesting characteristics, there is limited information about the genetic and molecular basis of ramie fiber yield (FY) and its related traits (including stem length (SL) and diameter (SD), bark weight (BW) and thickness (BT)^4–6^. Only one expanded SSR markers linkage map and one SNP markers linkage map have been developed in ramie, resulting in limited quantitative trait loci (QTLs) identified for FY and its related traits^6,7^. One BT QTL, *qBT4a*, has been ascertained its candidate gene that encodes a MYB transcription factor, based on the identification of an insertion of large-fragment that resulted in premature termination in this candidate gene in one parent^6^. However, the function of *qBT4a* has not been validated by further research.

Cultivated ramie is deemed to be domesticated from the wild progenitor *B. nivea* var. *tenacissima* (BNT) according to morphological, genetic and molecular evidences^8,9^. There are remarkable morphological differences between cultivated ramie and wild BNT, especially stem length and diameter^10^. The traits of stem length and diameter are two important components for determining the fiber yield of ramie, and their changes likely result from the domestication to meet human need. Recently, transcriptome comparison between ramie and BNT had identified several positively selected genes involved in the fiber yield^8^. Therefore, the yield-related traits have undergone principally selection in the ramie domestication by human. However, the genetic basis about the domestication of yield-related traits is still uncharacterized.

In the present study, to understand the genetic basis of yield-related traits and their domestication, we developed an F_2_ population derived from two parents, the cultivar Zhongsizhu 1 (ZSZ1) and wild progenitor BNT; and then, a high-density genetic map was constructed, and QTLs for yield-related traits were identified. This study provided insights into the genetic basis of fiber yield-related traits in ramie, and the identification of many QTLs presented a basis for improving the yield traits in ramie breeding.

## Results

### Variation of FY and its related traits in the population and two parents

The two parents, ZSZ1 and wild BNT exhibited highly significant difference in five fiber yield-related traits investigated (Table 1; Supplementary Fig. S1). ZSZ1 showed longer and thicker stems (130.5 cm in length and 9.11 mm in diameter) than BNT (only 60.5 cm in length and 4.58 mm in diameter; Table 1). In addition, for stem barks, the thickness and weight were 0.69 mm and 19.9 g in ZSZ1, which were thicker and more weight than BNT (0.42 mm in thickness and 2.6 g in weight; Table 1). Because all four traits above had important influence on of FY, the difference of them resulted in significant difference in FY in two parents (2.07 g and 0.25 g for ZSZ1 and BNT, respectively; Table 1). There were wide variations observed in the population for five investigated traits in the two environments (Table 1). ANOVA revealed that the genetic factor (δ_g_^2^) explained most observed phenotypic variance for all five traits in the population; except the BT trait, all traits had a high heritability in the population, ranged from 80.4% to 87.7% (Table 2). In addition, the five traits showed significant positive correlations (*P* < 0.01; Table 3).

**Table 1:** Descriptive statistics of the traits for the parents and the population.

Traits	Environment 1		Environment 2		Parents	
	Range	Mean ± SD	Range	Mean ± SD	ZSZ1	BNT
SD (mm)	4.18–11.73	8.56 ± 1.42	2.48–11.46	7.00 ± 1.50	9.11	4.58
SL (cm)	61.7–195.6	142.7 ± 23.5	35.8–158.3	111.4 ± 23.5	130.5	60.5
BW (g)	2.0–26.6	12.3 ± 5.3	1.0–25.0	10.0 ± 5.8	19.9	2.6
BT (mm)	0.27–0.70	0.46 ± 0.08	0.23–0.84	0.51 ± 0.13	0.69	0.42
FY (g)	0.26–4.42	1.87 ± 0.86	0.15–3.87	1.36 ± 0.85	2.07	0.25

**Table 2:** Variance estimation and heritability for five traits in the population.

Traits	δ_g_^2^	δ_ge_^2^	δ_e_^2^	Heritability (%)
SD	1.24	0.16	0.23	85.4
SL	282.02	26.54	123.01	83.1
BW	18.81	1.83	3.22	87.7
BT	0.049	0.020	0.011	68.6
FY	0.55	0.12	0.04	80.4

δ_g_^2^, δ_ge_^2^ and δ_e_^2^ indicate the genetic, interaction of genetic by environment, and error variances derived from the mean square expectations of the analysis of variance.

**Table 3:** Correlation coefficient of five yield-related traits.

	SD	SL	BW	BT	FY
SD		0.78*	0.78*	0.67*	0.85*
SL	0.86*		0.64*	0.53*	0.72*
BW	0.90*	0.82*		0.71*	0.89*
BT	0.80*	0.69*	0.78*		0.68*
FY	0.65*	0.61*	0.75*	0.53*	

*Significant correlation at 1%.

### High-density genetic map

**Figure 1:**
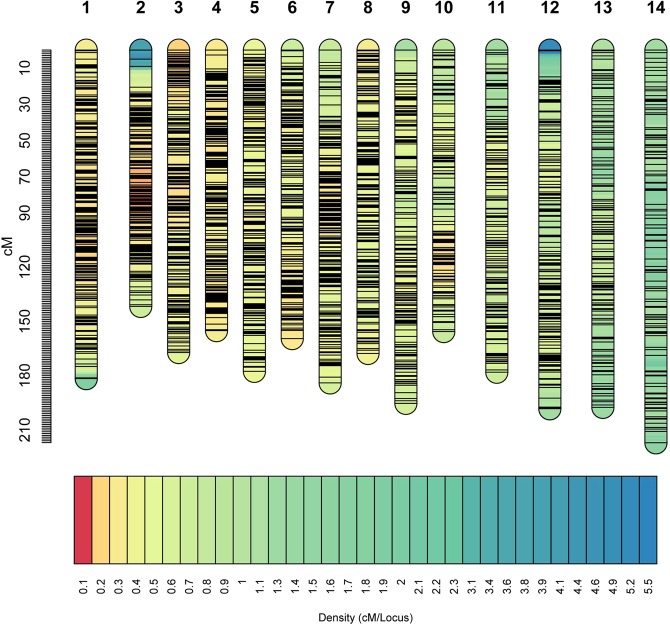
High-density genetic map developed using the population derived from cultivated ramie Zhongsizhu 1 and wild *B. nivea* var. *tenacissima*. The 2476.5-cM map includes 6,433 SNPs.

A total of 26,450 high-quality polymorphic SNPs, with the segregation pattern of aa × bb between two parents, have been identified. After filtering, 6,811 SNPs were performed for grouping, resulting in 14 linkage groups. Thereafter, the SNPs unassigned into linkage groups were filtered, resulting in a total of 6,433 SNPs used to develop a high-density genetic map (including 2,175 binned markers), with a total length of 2476.5 cM (Fig. 1). The length of individual linkage groups ranged from 141.7 to 217.1 cM. These 6,433 SNPs were distributed evenly in the genetic map; there were 108–196 SNPs contained in per linkage group, and the average and maximum distance of interval was 0.38 cM and a 14.35 cM, respectively (Table 4). Only 23 intervals had genetic distances of >5 cM (Table 4).

**Table 4:** Summary of the high-density genetic map.

Linkage group	Number of markers	Number of bin markers	Length (cM)	Average marker interval (cM)	Maximum interval (cM)	Number of interval with >5 cM
LG 1	762	196	181.7	0.24	6.34	1
LG 2	686	153	141.7	0.21	11.46	1
LG 3	685	184	167.2	0.24	4.97	0
LG 4	643	175	155.1	0.24	7.72	1
LG 5	507	177	177.6	0.35	4.52	0
LG 6	500	162	159.6	0.32	4.97	0
LG 7	496	180	184.0	0.37	6.80	3
LG 8	440	165	167.7	0.38	4.52	0
LG 9	404	151	195.3	0.48	6.80	3
LG 10	367	112	155.6	0.43	5.43	1
LG 11	291	132	178.1	0.61	5.88	3
LG 12	286	153	198.3	0.7	14.35	2
LG 13	203	127	197.5	0.98	6.80	2
LG 14	163	108	217.1	1.34	8.64	6
All	6,433	2,175	2,476.5	0.38	14.35	23

### QTL mapping for FY and its related traits

**Figure 2:**
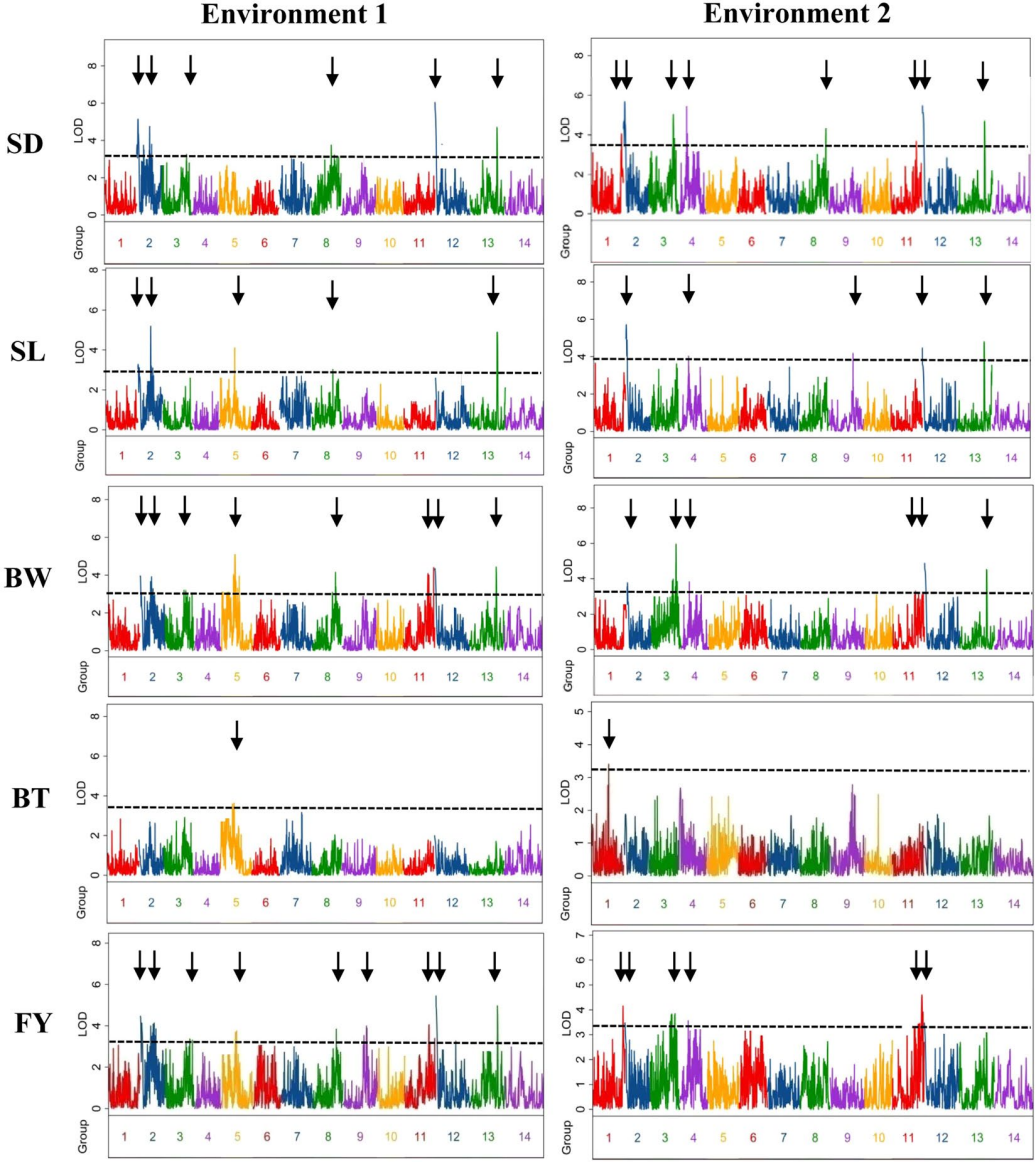
Quantitative trait loci (QTLs) for fiber yield and its related traits. The broken line indicates the genome-wide significance LOD threshold, and the black arrow indicates the LOD peak of the QTLs. The left and right figures indicate the genome-wide identification of QTLs for each trait in Environment 1 and Environment 2, respectively.

QTL analysis for five traits was performed by the program of Windows QTL CARTOGRAPHER. LOD thresholds (1000 permutations) were estimated for five investigated traits in two environments, and the result indicated that the values ranged from 3.3 to 3.8 (Table S1). Based on these thresholds, a total of 29 and 26 QTLs were identified for these five traits in Environment 1 and Environment 2, respectively, nine and twelve of which had an overdominance in corresponding environment (Table 5, Fig. 2). There were 16 QTLs detected in both two environments. Finally, in all 39 QTLs were identified, i.e., nine, eight, nine, two, and eleven QTLs for SD, SL, BW, BD and FY traits, respectively (Table 5, Fig. 2). Among these 39 QTLs, two SD QTLs (*SD2-1*, and *SD13*), three SL QTLs (*SL2-1*, *SL9* and *SL13*), two BW QTL (*BW2-1* and *BW13*), one BT QTL (*BT1*), and three FY QTLs (*FY2-1 FY9* and *FY13*) improve the trait by the wild BNT allele, whereas the others increase the phenotype by ZSZ1’s.

**Table 5:** QTLs identified for fiber yield and four its related traits from ZSZ1/BNT population by QTL CARTOGRAPHER program.

Traits	QTL	Linkage group	Environment 1						Environment 2					
			PP^a^	LOD	Add^b^	Dom^c^	D/A^d^	Var%^e^	PP^a^	LOD	Add^b^	Dom^c^	D/A^d^	Var%^e^
SD	*SD1*	1							171.8	4.06	−0.8	−0.6	0.8	9.6
	*SD2-1*	2	8.0	5.14	0.6	0.9	1.5	15.8	7.0	5.69	0.6	1.2	1.9	13.0
	*SD2-2*	2	73.2	4.75	−1.0	0.6	−0.7	14.5						
	*SD3*	3	136.1	3.47	−0.6	−0.5	0.9	9.9	139.3	5.03	−0.9	−0.1	0.2	11.7
	*SD4*	4							46.0	4.65	−1.0	0.1	−0.1	10.9
	*SD8*	8	139.7	4.42	−0.9	−0.1	0.1	13.3	139.7	4.82	−0.9	−0.2	0.2	11.2
	*SD11*	11							144.3	3.67	−0.5	−0.7	1.3	8.7
	*SD12*	12	2.5	5.46	−0.7	1.0	−1.5	17.0	2.5	5.15	−0.8	0.9	−1.2	11.9
	*SD13*	13	151.9	4.7	0.7	0.3	0.5	14.3	151.9	4.68	0.6	0.9	1.4	10.9
SL	*SL2-1*	2	2.0	3.56	7.8	13.6	1.8	13.1	2.0	5.71	7.6	20.6	2.7	21.8
	*SL2-2*	2	73.2	5.19	−16.5	12.0	−0.7	20.0						
	*SL4*	4							46.5	4.02	−13.9	−1.7	0.1	15.9
	*SL5*	5	83.4	4.11	−14.8	6.1	−0.4	16.2						
	*SL8*	8	139.7	3.55	−13.4	2.2	−0.2	12.1						
	*SL9*	9							136.7	4.17	13.7	3.8	0.3	16.4
	*SL12*	12							1.5	4.46	−6.5	17.4	−2.7	17.5
	*SL13*	13	152.9	4.9	12.9	11.5	0.9	18.9	152.9	4.8	9.8	20.2	2.1	18.7
BW	*BW2-1*	2	1.0	3.96	3.1	0.9	0.3	10.2	2.0	3.53	2.2	3.2	1.4	9.9
	*BW2-2*	2	77.7	3.83	−3.3	0.4	−0.1	9.9						
	*BW3*	3	136.1	3.86	−1.7	−2.9	1.7	9.9	139.3	5.96	−3.7	−0.6	0.2	15.8
	*BW4*	4							46.5	3.83	−2.9	−1.4	0.5	10.6
	*BW5*	5	83.4	5.31	−3.7	0.3	−0.1	13.3						
	*BW8*	8	129.8	4.15	−1.8	−2.9	1.6	10.6						
	*BW11*	11	139.3	4.08	−2.3	−2.5	1.1	10.5	135.2	4.01	−2.5	−2.4	1.0	11.1
	*BW12*	12	1.45	4.23	−2.7	2.0	−0.8	10.9	1.5	4.52	−2.8	2.6	−0.9	12.4
	*BW13*	13	151.9	4.43	2.7	1.0	0.4	11.2	151.9	4.32	2.4	3.3	1.4	11.7
BT	*BT1*	1							97.3	3.4	0.021	−0.026	−1.2	13.6
	*BT5*	5	76.7	3.62	−0.049	−0.0003	0.0	14.4						
FY	*FY1*	1							171.8	4.15	−0.6	−0.4	0.8	16.4
	*FY2-1*	2	1.0	4.41	0.5	0.2	0.4	8.7	1.0	3.47	0.6	0.1	0.3	13.9
	*FY2-2*	2	77.7	4.74	−0.6	0.1	−0.2	9.3						
	*FY3*	3	136.1	4.39	−0.4	−0.4	1.0	8.6	138.4	3.78	−0.6	0.0	0.0	15.0
	*FY4*	4							46.5	3.56	−0.5	−0.2	0.4	14.2
	*FY5*	5	83.4	3.75	−0.5	0.3	−0.6	7.5						
	*FY8*	8	139.7	4.13	−0.5	0.0	−0.1	8.2						
	*FY9*	9	133.5	4.16	0.5	−0.2	−0.5	8.2						
	*FY11*	11	139.3	4.05	−0.3	−0.5	1.6	8.0	144.3	4.56	−0.5	−0.5	1.1	17.8
	*FY12*	12	0.5	5.44	−0.3	0.6	−1.9	10.5	0.5	3.47	−0.5	0.3	−0.6	13.9
	*FY13*	13	151.9	4.96	0.4	0.3	0.8	9.6						

^a^LOD peak position. ^b^Additive effect, positive additive effect indicates that the BNT allele increased the trait. ^c^Dominance effect. ^d^The ratio of dominance effect to additive effect. ^e^Percentage of total phenotypic variance explained by the QTL.

Additionally, QTLs for these five traits were also detected by the QTL.gCIMapping.GUI program. There were six, four, four, and eight QTLs identified for SD, SL, BW, and FY trait, respectively; however, no QTL of BT trait was detected (Table 6). Among these 22 QTLs, 14 have been mapped by Windows QTL CARTOGRAPHER, and 9 improve the traits by the wild BNT allele (Table 6). Finally, a total of 47 QTLs were identified for five traits by two programs of QTL analysis, of which 14 increase the trait phenotype by wild BNT allele.

**Table 6:** QTLs identified for fiber yield and four its related traits from ZSZ1/BNT population by the QTL.gCIMapping.GUI program.

Traits	QTL^a^	Environment	Linkage group	QTL position	LOD	Add^b^	Var%^c^
SD	*SD2-1*	2	2	1.0	3.39	0.5	4.5
	*SD3-1*	2	3	18.0	4.30	0.5	3.2
	*SD3*	2	3	141.1	2.54	−0.3	1.8
	*SD4*	2	4	47.8	2.51	−0.3	2.4
	*SD6*	2	6	142.9	5.21	0.7	5.2
	*SD11*	2	11	147.0	3.96	−0.5	2.6
SL	*SL2-1*	2	2	1.0	3.91	9.1	3.7
	*SL8*	2	8	139.7	2.71	−6.0	3.3
	*SL13*	1	13	151.9	3.47	7.6	5.3
	*SL13-1*	2	13	197.5	2.82	−7.3	2.4
BW	*BW2-1*	2	2	2.0	3.37	2.1	6.7
	*BW3-1*	2	3	18.0	2.78	1.8	2.5
	*BW5*	1	5	83.0	3.31	−1.4	3.6
	*BW13*	1	13	151.9	4.93	1.8	5.8
FY	*FY1-1*	2	1	33.8	3.06	−0.1	1.7
	*FY2-1*	2	2	0.0	15.98	0.6	14.9
	*FY3*	2	3	141.0	4.37	−0.2	2.1
	*FY4-1*	2	4	114.0	3.09	−0.1	1.7
	*FY5*	2	5	80.4	3.06	−0.2	2.1
	*FY10*	2	10	149.0	12.23	−0.5	12.9
	*FY11*	2	11	143.0	7.22	−0.4	5.4
	*FY14*	2	14	32.0	2.94	−0.2	2.1

^a^QTL underlined indicates it be detected by both programs; ^b^Additive effect, positive additive effect indicates that the BNT allele increased the trait; ^c^Percentage of total phenotypic variance explained by the QTL.

### QTL cluster for fiber yield-related traits

Among 47 QTLs, 40 were located in 12 pleiotropic genomic regions, thus constituting 12 QTL clusters (Table 7). Except Cluster 5, all clusters were made up of one FY QTL and one/several QTLs of FY-related traits (Table 7). Because the FY is determined by its related component traits, the FY QTL and fiber yield-related QTLs of each cluster potentially were one same pleiotropic QTL. Interestingly, in each cluster, all QTLs increased the traits phenotype by the alleles from the same parent (Table 7). Therefore, among 12 clusters, eight improve the FY and its related traits by ZSZ1 allele, and the other four by the allele of wild BNT. All QTLs of Cluster 10 displayed overdominance, indicating that this cluster is a genomic region with overdominance (Table 7).

**Table 7:** QTL clusters identified for five yield traits investigated.

QTL cluster	Linkage group	QTLs					Excellent allele	QTLs with overdaminance effect
		SD	SL	BW	BT	FY		
Cluster 1	1	*SD1*				FY1	ZSZ1	None
Cluster 2	2	SD2-1	SL2-1	*BW2-1*		*FY2-1*	BNT	*SD2-1*, *SL2-1*, and *BW2-1* (*BW2-1* only in Environment 2)
Cluster 3	2	SD2-2	SL2-2	*BW2-2*		*FY2-2*	ZSZ1	None
Cluster 4	3	*SD3*		*BW3*		*FY3*	ZSZ1	*BW3* and *FY3* (both in Environment 1)
Cluster 5	3	*SD3-1*		*BW3-1*			BNT	—
Cluster 6	4	*SD4*	*SL4*	*BW4*		*FY4*	ZSZ1	None
Cluster 7	5		*SL5*	*BW5*	*BT5*	*FY5*	ZSZ1	None
Cluster 8	8	*SD8*	*SL8*	*BW8*		*FY8*	ZSZ1	*BW8*
Cluster 9	9		*SL9*			*FY9*	BNT	None
Cluster 10	11	*SD11*		*BW11*		*FY11*	ZSZ1	All
Cluster 11	12	*SD12*	*SL12*	*BW12*		*FY12*	ZSZ1	*SD12*, *SL12*, and *FY12* (*FY12* only in Environment 1)
Cluster 12	13	*SD13*	*SL13*	*BW13*		*FY13*	BNT	*SD13*, *SL13*, *BW13* (all in Environment 2)

## Discussion

### Genetic Characterization of FY and its related traits

Ramie is an important industrial fiber crop, and the fiber yield and its four related traits are five of the most valuable traits in ramie production. To characterize the genetic basis of these traits, this study developed a high-density genetic map using 6,433 SNP marker. Based on this genetic map, there were 47 QTLs identified for FY and its related traits, 40 of which were mapped into the 12 genomic regions resulting in 12 QTL clusters. It is notable that significant positive correlations among FY and four its related traits were observed in the population. Therefore, we deduced that the existence of many QTL clusters was the genetic basis of the correlation of fiver traits investigated, which is accordance with the previous studies^7,11,12^.

Overdominance was deemed to be one of the most important reasons caused the heterosis for FY-related traits in ramie^7^. In this study, among 29 and 26 QTLs identified in Environment 1 and Environment 2 by Windows QTL CARTOGRAPHER, nine and twelve were found to have overdominance in corresponding environment, respectively. Interestingly, all three QTLs in Cluster 10 showed overdominance, suggesting that this pleiotropic regions might be the heterotic loci. In addition, there were seven QTLs displayed overdominance in single environment, which indicated that the overdominance effect can be affected by environment. Therefore, this study improved our understanding into the genetic basis of FY and FY-related traits.

### Insights into the genetic basis of the domestication of FY-related traits

Cultivated ramie is domesticated from the wild progenitor BNT^9^. Unlike cultivated ramie which shows outstanding performances in FY and FY-related traits, the BNT displayed inferior FY-related traits, and thus few fibers can be harvested from its stem barks. FY and FY-related traits were domesticated potentially by human in ramie. However, the genetic basis of the domestication for these traits has uncovered. In this study, we used a population derived from parent cultivated ZSZ1 and wild progenitor BNT to dissect the genetic basis of FY and FY-related traits, resulting in 47 QTLs.

There were 13 genes underwent positive selection identified by transcriptomic comparison between cultivated ramie and wild BNT^8^, of which two (CL10581Contig1 and T3_Unigene_BMK.28528) fell into the QTL region, and two (CL16310Contig1 and CL12943Contig1) were mapped into the regions near to the QTLs by aligning these transcripts to genome (Table S2). Among these four genes, CL12943Contig1 and T3_Unigene_BMK.28528 are two transcripts near to the *BT1* and Cluster 8, respectively, and their encoding-proteins WAT1-related protein and homeobox-leucine zipper protein have important roles in fiber differentiation and secondary wall thickening^13–15^. In addition, CL16310Contig1 encodes an ankyrin repeat-containing protein which is involved in the regulation of cell differentiation and development^16^, suggesting that this gene have a potential role in the stem growth; and CL16310Contig1 was found in the regions near to the Cluster 9. Therefore, these genes underwent positive selection potentially have important roles in the regulation of fiber yield. In this study, there were 10 QTLs (including nine QTLs from three clusters) identified to be near to these four genes. The adjacency between identified QTLs and positively selected genes provided new insights into the domestication of FY and FY-related traits.

### QTLs for ramie high-yield breeding

Improving the FY is one of the most important objectives in ramie breeding program. Understanding the genetic basis of FY and its related traits will be helpful for the improvement of these traits. In this study, a total of 47 QTLs were identified, and 40 QTLs were found to clustered distribute in 12 pleiotropic loci, including one heterotic loci. Thus, the application of these QTL clusters can improve the performance of several FY-related traits simultaneously by marker assisted selection. Among 47 QTLs, there were still 14 QTLs (including 12 QTL from 4 clusters) which increased phenotype of FY and its related traits by the alleles of wild BNT, although the wild BNT displayed inferior performance in these traits. In crop domestication, there are some alleles lost in some loci, including excellent alleles, which causes that the genetic diversity of traits dramatically drops^17–19^. Discovery and application of excellent gene resources from wild species were frequently performed by crop breeder. Therefore, the identification of 14 QTLs with excellent alleles from wild species was invaluable gene resources for the ramie high-yield breeding.

## Materials and Methods

### Experimental populations and investigation of traits

A population consisting of 111 F_2_ progenies was developed using two parents, the cultivar Zhongsizhu 1 (ZSZ1) and wild species *B. nivea* var. *tenacissima* (BNT), according to a previously described strategy^7^. Then, all 111 F_2_ progenies and two parents was reproduced by cutting propagation and generated an agamous line, which had the identical genotype to the corresponding F_2_ individual. All these agamous family lines and parents were grown in the experimental farm of the Institute of Bast Fiber Crops (IBFC) in June 2016. Two replications were grown in a randomized complete block design. For each family line, ten seedlings were grown into a plot of two rows, with a distance of 45 cm between rows and 70 cm between plants within a row. The area around the population was planted with two-row ramie which was used to eliminate the boundary effect in the population.

Ramie is perennial, and the overground part of plants can grow again when harvested. In this study, the population was collected phenotype in two growing environments, i.e. Environment 1 from Apr. 10, 2017 to June 14, 2017 and Environment 2 from June. 15, 2017 to Aug. 4, 2017., Population phenotype was collected when more than two-thirds leaves of ramie from the population had shattered. The stem length (SL) was measured from the stem bottom to the shoot. The stem diameter (SD) and bark thickness (BT) were measured in the middle of stem using a Vernier caliper. The stem bark was harvested individually and weighted, and bark weight (BW) was calculated as the mean bark weight per stem; and then, the fibers were extracted from these stem bark, and were dried, and then, the trait of fiber yield (FY) was estimated as the mean weight of fiber per stem.

### Genotyping of population

Young leaves which were individually collected from each line and parent were frozen in liquid nitrogen immediately, and stored at −80 °C until used. Total RNAs of each sample was extracted by the EZNA. Plant RNA Kit (OMEGA Bio-Tek, Norcross, GA, USA). Thereafter, these RNAs were used to construct a cDNA library with fragment lengths of ~250 bp. Paired-end sequencing was performed by the Illumina HiSeq. 2500 sequencing platform (Illumina, San Diego, CA, USA), at Shanghai OE Biotech. Co., Ltd (Shanghai, China). Finally, the raw reads were trimmed the adapter sequences and filtered low-quality reads, result in high quality clean reads (SRA accession no. SRP182925).

To genotype the population, the clean reads from the transcriptome of 111 F_2_ families and two parents were aligned into the ramie genome (NCBI accession no: PHNS00000000)^20^, using the software Burrows-Wheeler Aligner (BWA) with the default parameters^21^. The software SAMtools^22^ was used to convert the alignment files as bam files. If there are multiple read pairs with identical external coordinates, we retained the pair with the highest mapping quality.

Thereafter, SNPs were identified from the two parents and 111 families using the software SAMtools^22^, only high quality SNPs (coverage depth > 10, quality value > 50, minor allele frequency >_0.05, and missing genotype rate <_0.1) were retained. Because both the parent ZSZ1 and BNT were heterozygous, the polymorphic markers between them were detected according to the following steps: firstly, all polymorphic markers classified into eight segregation patterns, i.e., ef × eg, nn × np, ab × cc, aa × bb, ab × cd, lm × ll, hk × hk, and cc × ab^23,24^, according to the CP model in JoinMap 4.0^25^; Then, the SNPs with more than two genotypes were filtered out, finally retaining only polymorphic SNPs with the segregation pattern of ‘aa × bb’.

### Construction of genetic map

The genetic map was constructed according to the description of Liu *et al*.^6^. Firstly, Markers that contained abnormal bases or exhibited significantly distorted segregation (P < 0.001) or non-integrity (missing data in > 30% progenies) were filtered out using LepMap3^26^. Thereafter, the regression algorithm, three times circulation sequence, and Kosambi mapping function were used for marker distance calculation^27^, and the linkage map was constructed by the software LepMap3^26^, and was drawn using the mapchart 2.32^28^, with the default parameters.

### Data analysis

QTL analysis was performed according to the description of Liu *et al*.^6^. QTLs for fiber yield-related traits in two environments were detected using composite interval mapping (CIM) with Windows QTL CARTOGRAPHER2.5^29^. Window size was set at 10 cM, and forward stepwise regression was used to identify significant markers as cofactors. The experiment-wise LOD threshold significance level was determined by computing 1000 permutations (P < 0.05) using a permutation test program in Windows QTL CARTOGRAPHER2.5^29^. These permutations can account for non-normality in marker distribution and trait values. By comparing with the CIM method, the genome-wide composite interval mapping (GCIM) based on the multi-locus mixed model has high power in the detection of small and linked QTLs^30^, and thus, the QTL mapping for five traits was also performed by the QTL.gCIMapping.GUI program^31^, and an LOD threshold of 2.5 was set. For one same trait, if QTLs detected from different environments or by different programs are mapped within a 10-cM region, these QTLs were considered as a same QTL. If there were several QTLs of different traits identified into a 10-cM region, this region was deemed to be a QTL cluster.

ANOVA was performed by the program STATISTICA 8.0. Heritability was estimated according to the formula as follows:

$${{\rm{H}}}^{{\rm{2}}}=\frac{{{\sigma }_{g}}^{2}}{{{\sigma }_{g}}^{2}+{{\rm{n}}}^{-{\rm{1}}}{{\sigma }_{ge}}^{2}+{(\mathrm{nr})}^{-{\rm{1}}}{{\sigma }_{e}}^{2}},$$

where σ_g_^2^, σ_ge_^2^ and σ_e_^2^ were the estimates of genetic, genetic by environment interaction and error variances derived from the mean square expectations of the analysis of variance, with n = 2 being the number of environments and r = 2 being the number of replicates. Expected genotypic variance and expected genetic by environment interaction variance were estimated according to the model as the description of Liu *et al*.^32^.

## Supplementary information

Fig. S1, Table S1, Table S2

## Data Availability

The datasets generated for this study can be found in the NCBI SRA database under the accession number SRP182925.
