# Supplementary material for: Linkage mapping of quantitative trait loci for fiber yield and its related traits in the population derived from cultivated ramie and wild B. nivea var. tenacissima
Source: Sci Rep. 2019 Nov 14;9:16855. doi: 10.1038/s41598-019-53399-5 (PMC6856109; doi:10.1038/s41598-019-53399-5)
Supplement: Supplementary file 1 — Fig. S1, Table S1, Table S2 [file 41598_2019_53399_MOESM1_ESM.pdf]

Linkage mapping of quantitative trait loci for fiber yield and its related traits in the population derived from cultivated ramie and wild *B. nivea* var. *tenacissima*

Zheng Zeng[1†], Yanzhou Wang[1†], Chan Liu[1], Xiufeng Yang[2], Hengyun Wang[2], Fu Li[1], Touming Liu [1]*

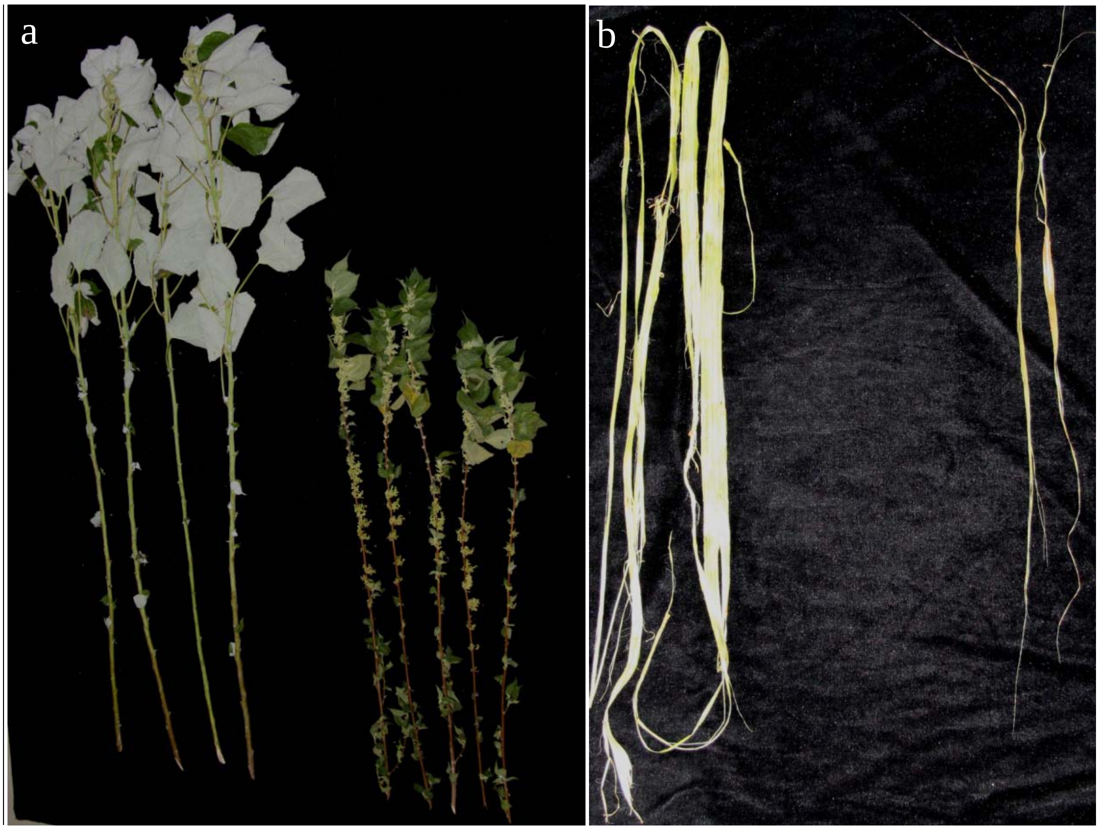

Fig. S1 Comparison of the stem morphology (a) and bast fiber (b) in two parents. Left and right stems in a figure derive from ZSZ1 and BNT, respectively; and left and right fibers in b figure are extracted from the stem of ZSZ1 and BNT, respectively.

Table S1 LOD thresholds determined by computing 1,000 permutations ($P < 0.05$).

	SD	SL	BW	BT	FY
Environment 1	3.3	3.5	3.8	3.5	3.6
Environment 2	3.6	3.5	3.5	3.3	3.4

Table S2 Mapping of 13 positively selected genes reported previously to genetic map

Positively selected unigene		Unigene position		The closest marker			QTL cluster/QTL near to unigene	
ID	Annotation	Scaffold	Position (bp)	Marker	Linkage group	Position (cM)	Cluster	Interval
CL10581Contig1	Desiccation-related protein	PHNS01004728.1	2666492-2669372	Bni_Flc_00155	3	139.3	Cluster 4	136.1 cM -139.3 cM
CL16310Contig1	Ankyrin repeat-containing protein	PHNS01001832.1	1857397-1858592	Bni_Flc_30664	9	139.8	Cluster 9	133.5 cM-136.7 cM
CL155Contig1	cc-nbs-lrr resistance protein	PHNS01010760.1	98253-101117	Bni_Flc_23241	5	110.4		
CL1901Contig1	LRR and NB-ARC domains-containing disease resistance protein	PHNS01008711.1	1053205-1055272	Bni_Flc_27766	8	52.3		
CL17Contig4	Putative disease resistance protein	PHNS01008711.1	66221-64235	Bni_Flc_07667	8	58.6		
T2_Unigene_BMK.26411	GEM-like protein	PHNS01012043.1	381150-382432	Bni_Flc_21457	2	33.1		
CL4554Contig1	Mitogen-activated protein kinase kinase	PHNS01006314.1	3600044-3598842	Bni_Flc_16544	3	76.6		
CL17097Contig1	Myb family transcription factor	PHNS01007792.1	2672141-2670992	Bni_Flc_07833	6	95.5		
T3_Unigene_BMK.28528	Homeobox-leucine zipper protein	PHNS01012126.1	3778193-3777442	Bni_Flc_20772	8	139.7	Cluster 8	129.8 cM-139.7 cM
CL12943Contig1	WAT1-related protein	PHNS01008720.1	189859-190718	Bni_Flc_27035	1	94.6	*BT1*	97.3 cM
CL7774Contig1	Gibberellin 2-beta-dioxygenase	PHNS01005842.1	7190032-7185705	Bni_Flc_18944	9	167.3		
CL5156Contig1	WEB family protein	PHNS01004769.1	959381-963103	Bni_Flc_20626	8	91.0		
CL6432Contig1	3-hydroxyisobutyryl-CoA hydrolase-like protein	PHNS01002761.1	6107-10034					
